# Iron Deficiency and Deranged Myocardial Energetics in Heart Failure

**DOI:** 10.3390/ijerph192417000

**Published:** 2022-12-18

**Authors:** Michał Tkaczyszyn, Krzysztof Michał Górniak, Weronika Hanna Lis, Piotr Ponikowski, Ewa Anita Jankowska

**Affiliations:** 1 Institute of Heart Diseases, Wroclaw Medical University, 50-556 Wroclaw, Poland; 2 Institute of Heart Diseases, University Hospital, 50-566 Wroclaw, Poland

**Keywords:** iron deficiency, myocardial tissue, energy generation, metabolic derangements

## Abstract

Among different pathomechanisms involved in the development of heart failure, adverse metabolic myocardial remodeling closely related to ineffective energy production, constitutes the fundamental feature of the disease and translates into further progression of both cardiac dysfunction and maladaptations occurring within other organs. Being the component of key enzymatic machineries, iron plays a vital role in energy generation and utilization, hence the interest in whether, by correcting systemic and/or cellular deficiency of this micronutrient, we can influence the energetic efficiency of tissues, including the heart. In this review we summarize current knowledge on disturbed energy metabolism in failing hearts as well as we analyze experimental evidence linking iron deficiency with deranged myocardial energetics.

## 1. Introduction

Heart failure (HF) is one of the leading challenges for healthcare systems worldwide due to the growing patient population, frequent re-hospitalizations and finally the low quality of life of patients, often with multimorbidity and/or frailty [1]. Adverse metabolic myocardial remodeling as reflected by abnormal energy generation constitutes the hallmark of HF pathophysiology, and translates into further progression of both ‘central’ myocardial dysfunction and ‘peripheral’ organ maladaptations (such as of adipose tissue or skeletal muscles) [2,3,4,5,6]. Impaired energy production and utilization affecting the heart and other organs is considered not only an important pathophysiological feature of disrupted homeostasis occurring during circulatory decompensation but also an emerging therapeutic target during later long-term clinical recovery/improvement or not [7,8].

Energetic impairment of cardiac muscle and other organs can contribute to recurrent episodes of worsening HF along with inevitable entering the path of a terminal, end-stage phase of the disease [9,10]. There is experimental evidence that iron deficiency (ID) is linked with energy depletion within myocardial and other tissues (such as skeletal muscles) [11,12,13]. Of note, it has been demonstrated that intravenous iron therapy with ferric carboxymaltose (FCM) administered after an episode of acute HF in patients with systemic ID (evaluated based on circulating iron parameters) can prevent recurrent HF hospitalizations [14]. The intriguing, hypothetical concept of affecting/improving myocardial energy metabolism using particular substances given orally or intravenously (such as iron formulations) requires further translational research, in particular because myocardial iron status (strictly in the heart) is not comprehensively studied [15]. It is worth mentioning that we have limited data suggesting possible improvement of skeletal muscle energetics due to iron therapy in HF patients with ID [16]. In the current review paper, we discuss the hypothesis of whether intravenous iron repletion may have clinically beneficial effects through improving myocardial energetics in iron-deficient patients with HF.

## 2. Physiology of Cardiac Energetics

Although the heart utilizes a lot of energy, the reserve of adenosine triphosphate (ATP) and biofuels within this organ is limited [3,17,18]. Myocardial tissue is characterized by potent oxidative capacity and ATP generation, mainly due to a high mitochondrial density and a high efficacy of citric acid cycle (CAC) enzymes [4]. More than 95% of ATP generated in the heart originates from highly efficient mitochondrial oxidative phosphorylation [17]. The turnover of high-energy phosphates in the myocardium is very dynamic and efficient—although cardiac ATP stores amount to less than 1.0 g, the heart utilizes as much as a few kilograms daily [19]. Regarding biofuels, myocardial tissue is considered ‘omnivorous’, and cardiomyocytes utilize predominantly fatty acids (FAs), glucose and lactate, with the limited use of amino acids and ketones [17,20,21]. Under physiological conditions, the β-oxidation of FAs accounts for the majority of produced cardiac acetyl-coenzyme A, a common compound synthesized also from other energy substrates [17]. The role of the creatine kinase (CK) system needs also to be mentioned here [2,3]. During extensive muscle performance with high ATP turnover, phosphocreatine (PCr) donates the high-energy phosphate bond in the reaction catalyzed by the myofibrillar CK isoenzyme, and the high temporal ATP concentration can be achieved [2].

Complex enzymatic repertoire allocated in the mitochondrial matrix and membranes enables these organelles to play the fundamental role in the efficient oxidative metabolism within the myocardial tissue [4,22,23,24]. Mitochondria account for almost one third of the cardiac muscle tissue volume, and as mentioned above, the vast majority of energy generated in cardiomyocytes originates from mitochondrial respiration [23,25]. Mitochondria are not only cellular ‘power plants’, but are also involved in the processes of cardiomyocyte apoptosis, calcium handling and reactive oxygen species (ROS) generation [23,26].

The regulation of myocardial energy metabolism is not only based on direct modulation of individual metabolic pathways, but is also influenced by hormones (e.g., thyroid, pituitary or steroidal), and responds dynamically to the supply of particular energy substrates [2,6,17,27,28,29]. Adenosine monophosphate-activated protein kinase (AMPK) is an example of master energy and redox sensor, which in response to diverse stress stimuli for the cardiomyocyte (not only energy deficit but also, for example, excessive oxidative stress) plastically modulates intracellular metabolism to promote catabolic pathways crucial for survival at the expense of temporarily less important anabolic ones [30]. As mentioned above, the fasted vs. fed state can also impact myocardial substrate preference. Starvation is characterized by low circulating insulin, accompanied by down-regulated sarcolemmal glucose transporters proteins, augmented lipolysis and high circulating FAs, which results in the preferential utilization of FAs through β-oxidation [17,29,31].

## 3. Deranged Energy Metabolism in HF

### 3.1. Adverse Metabolic Myocardial Remodeling in HF

Traditionally, the term ‘myocardial remodeling’ has been dedicated to describe the structural and functional changes (firstly adaptive but later maladaptive) occurring in a damaged heart [32,33]. In recent two decades, however, this term has acquired a new meaning, as energetic and mitochondrial disturbances occurring in failing heart have also been considered as an element of ‘myocardial remodeling’, and even the term ‘metabolic remodeling’ has been proposed in this context [5,6,27].

The prominent metabolic feature of a cardiac failure is energy starvation due to either inefficient energy production or/and increased energy demand (promoted by increased preload or/and afterload, tachycardia or sympathoexcitation) [2,4,27]. This view has been strongly supported by both experimental evidence coming from animal models of cardiac hypertrophy or HF of different aetiology [2,4,27,33], and clinical data arising from studies performed in vivo in humans based on the metabolomic approach [34,35]. Indeed, failing hearts are characterized by abnormal profile of high-energy phosphates and most prominent abnormalities are detected in advanced HF [2,4]. In particular, myocardial tissue from patients and animals with advanced HF is characterized by a reduced ATP content up to 40% [36,37,38]. In a few available studies (human studies or animal models) it has also been demonstrated that cardiac dysfunction in the course of HF is accompanied by depleted amount of both creatine and PCr, decreased CK activity, altered CK isoform distribution, and the down-regulated creatine transporter [37,39,40,41,42]. Another prominent characteristic of failing myocardium is a shift towards the glucose utilization as the primary substrate for energy generation [43,44,45]. The role of this shift is to minimize the oxidative loss occurring during the FAs utilization, and to increase the ATP generation/oxygen consumption ratio [43,44,46]. The increased glucose utilization via the glycolytic pathway is induced by different stimuli such as reduced oxygen supply, AMPK activation or depleted FAs metabolism [27]. In the early stages of HF, the FA utilization in myocardium may be unchanged or only slightly altered [17,47]. However, finally, several factors contribute to the diminished use of FAs and in the advanced stage of the disease their oxidative metabolism is markedly depleted [17,27,48]. It is worth mentioning that some authors rank altered substrate metabolism and oxidative stress within the myocardium higher than net deficiency/inefficient regeneration of high-energy phosphates in terms of the importance for adverse metabolic remodeling of the failing heart [5]. Importantly, in the course of HF diverse energetic maladaptations within myocardial tissue are promoted by increased sympathetic drive and overactive renin-angiotensin-aldosterone system, which further augment oxidative stress and are responsible for disordered calcium handling [18]. Moreover, the excessive adrenergic signaling accompanying the progression of HF, along with the subsequent insulin-resistance [49], have detrimental impact on the primarily adaptive shift towards glucose metabolism, as they decline the intracellular glucose utilization and increase lipotoxic properties associated with high circulating FAs [2,5,29,50,51,52,53]. Not without significance in the context of adverse myocardial metabolic remodeling are also not fully established myocardial effects of excessive visceral/epicardial adiposity and/or systemic disorders of carbohydrates metabolism [54,55].

Importantly, there is no clear borderline between the adaptive reversible stage and the maladaptive irreversible stage of metabolic derangements and mitochondrial dysfunction accompanying the natural history of myocardial dysfunction. In the earlier stage, all these metabolic derangements are presumed to play an adaptive role within viable but partially damaged or stunned cardiomyocytes, and in this situation therapeutic interventions ameliorating these pathologies seem to be at least feasible. In the latter stage, the progression of metabolic derangements accompanied by multifaceted mitochondrial dysfunction are rather maladaptive, and when persisted—lead inevitably from overt to end-stage HF, where any attempts to reverse these pathologies seem to be less or even not effective [6,10,33]. 

### 3.2. Mitochondrial Dysfunction in the Failing Heart

Accumulated evidence from basic science studies and animal models indicate the key role of impaired mitochondrial biology for the pathophysiology of HF [22,23,24]. Mitochondrial dysfunction is considered to represent the advanced stage of adverse myocardial remodeling [22,27,56,57]. There has been proposed a hypothesis that the occurrence of mitochondrial insufficiency is the critical point in the transition from compensated cardiac hypertrophy to overt HF [28,33], however, abnormal myocardial energetics and mitochondrial dysfunction have been found already in patients with cardiac hypertrophy [58]. In the course of HF, myocardial mitochondria are characterized by distinct structural alterations, impaired biodynamics, increased ROS generation as well as functional abnormalities consistent with inefficient oxidative capacity (of separate mitochondria themselves and of failing myocardium globally) [22,23,33,57]. In one study comparing left ventricular samples from advanced HF patients (referred for heart transplantation) versus donors without HF, Melenovsky et al. have demonstrated, firstly, decreased myocardial mitochondrial oxidative metabolism in HF, and secondly, that some of these mitochondrial derangements within failing hearts were related to myocardial ID (see further paragraphs) [59]. While the key element promoting the progression to end-stage HF (and hypothetically a therapeutic target to prevent this deterioration) is naturally believed to be the failure of the mitochondria to produce enough energy (‘mismatch’ between energetic demand and supply), it should be noted that other mitochondria-related pathologies can also contribute to lethal cardiomyopathy, such as uncontrollable ROS generation, abnormal functioning of heat shock proteins, altered mechanisms of apoptosis or dysregulated Ca^2+^ dynamics [22,57,60,61].

## 4. Iron and Energy Metabolism in the Heart

### 4.1. Beyond Circulating Biomarkers—Pools of Iron

**Figure 1:**
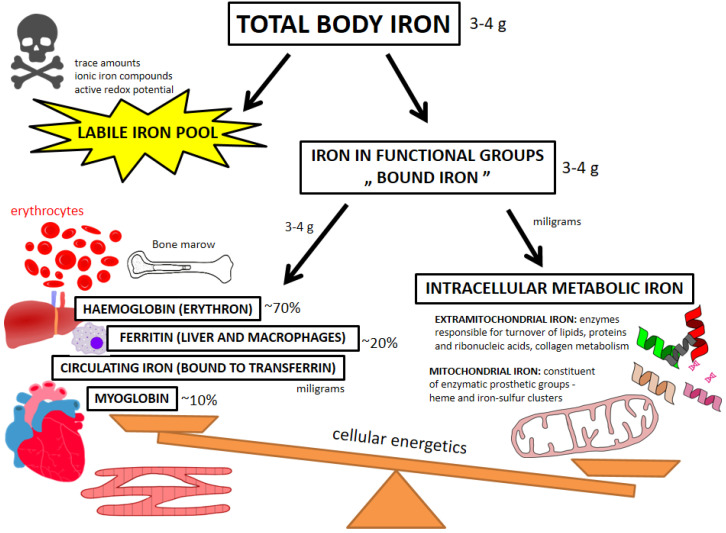
Iron pools in the body with special emphasis on physiological roles.

Human body contains approx. 3–4 g of iron in total, with the majority (~70%) in the erythron, ~20% being stored in the liver or mononuclear phagocyte system cells, ~10% incorporated in muscular myoglobin and only single milligrams circulating [62,63] [Figure 1]. It needs to be emphasized that just another milligrams of iron make up the total amount of the “bioactive” and metabolic pool, which is present in virtually all cells within the organism [62,63,64,65]. Due to unique chemical properties of two oxidative states, iron has a number of diverse biological functions—as the constituent of enzymatic machineries, intracellular energetic centers within mitochondria, and molecular transportation systems—in both hematopoietic and non-hematopoietic tissues [63,64,65]. Systematically, we should distinguish labile iron pool (LIP) and bound iron (within functional groups) [64,66]. LIP are basically redox active ionic iron compounds which are capable of inducing deleterious oxidative stress to the cell [24,64,65,66]. Not surprisingly, this potentially harmful pool is only responsible for a trace amount of iron in the body and an elegant repertoire of diverse molecules involved in the transportation, handling and storage of iron maintains LIP at the lowest possible level sufficient for cell physiology [64,65,66]. Consequently, almost all body iron is encompassed by the iron in functional groups, which is not directly harmful for the cells. Chemically, iron is an element of two major groups of proteins—hemoproteins (e.g., hemo- and myoglobin, catalases, [per]oxidases and cytochromes) and non-heme iron-containing proteins (e.g., aconitase or ferredoxin) [62,63,64,65]. In general, bound iron resources ‘by weight’ are related to the characteristics of particular organs/tissues where we can further distinguish storage-transportation and functional-metabolic pools of this nutrient [62,63,64,65] (Figure 1). With the majority of body iron being present in the erythrocytes and their bone marrow precursors (as the element of hemoglobin), other organs containing significant amounts of this nutrient are liver (where iron is stored in ferritin in parenchymal cells) and myocardial and skeletal muscle tissue (where iron is mainly represented as the constituent of myoglobin) [63,65].

Importantly, iron is also deposited and recycled by the mononuclear phagocyte system macrophages as well as it constantly circulates in blood bound to transferrin, with the latter acting as a supplier of iron for dedicated tissues [63,64]. Functional-metabolic pool of iron refers to iron incorporated into specialized extra-mitochondrial and mitochondrial enzymatic machineries and proteins involved in diverse physiological functions. Outside mitochondrial energetic machinery iron serves as a component of enzymes related to the turnover of lipids, proteins and ribonucleic acids, and is involved in the metabolism of e.g. collagen or amino acids [65,67,68,69,70,71,72,73]. In mitochondria, iron is an important constituent of enzymatic prosthetic groups—heme and iron-sulfur clusters. Both these prosthetic groups play a role in the mitochondrial energy generation through oxidative phosphorylation (as the components of the respiratory chain) [71,72,73].

The human body cannot actively remove excessive iron in case of overload and therefore iron absorption is tightly controlled, in particular through the effects of hepatic peptide hormone hepcidin [62,65,74]. Highly specialized transporters and receptors are variously expressed in particular tissues to maintain precise uptake and release of iron such as ferroportin (duodenum or mononuclear phagocyte system cells), transferrin receptor, haephastin or emerging iron regulatory proteins (IRP) which modulate the expression of iron homeostasis genes [75]. Importantly, since both LIP is toxic and ID detrimental for cell homeostasis, there is an evident U-shaped relationship between the amount of iron and cell death [76,77,78]. Indeed, in experimental animal models the states of both depleted and excessive iron are characterized by the marked mitochondrial dysfunction and increased oxidants generation [76,77,78]. This is also why some authors express their concerns related to intravenous iron therapy that may potentially lead to the accumulation of redox-active iron in cells and consequently result in severe oxidative stress [79]. There are well-known genetic disorders characterized by iron overload confirming that excessive accumulation of iron is harmful to various organs [80]. On the other hand, ID can also generate increased oxidative stress within the cell due to impaired anti-oxidative defense [81]. In one study already cited above the authors analyzed comprehensive cardiomyocyte mitochondrial functioning in relation to myocardial iron content in explanted failing human hearts, and have shown that myocardial ID correlated not only with reduced activity of CAC enzymes, but also with reduced expression of proteins responsible for ROS defense [59].

Owing to the critical role of iron for cellular oxidative metabolism and energy generation, ID is particularly detrimental for tissues with enhanced synthesis of various compounds (such as the liver or the kidneys) or those characterized by high energy demand [64,82]. Liver may be an example here—mice lacking iron regulatory proteins in the liver develop ID within mitochondria, which is followed by an abnormal mitochondrial metabolism of prosthetic groups containing iron, and this leads to lethal failure of the organ [73]. Skeletal muscle tissue is also very sensitive to impaired iron homeostasis [83,84,85,86]. Limited but analogous data have been provided for the heart—for example, the induction of dietary ID in experimental animals is followed by mitochondrial abnormalities in the myocardial muscle (e.g., mitochondrial swelling, structural sarcomere alterations, increased mitochondrial cytochrome c release) along with the development of hypertrophy and dilatation of left ventricle [12].

### 4.2. ID in the Heart—Clinical and Experimental Evidence

In recent years, there has been an increase in interest in ID as a comorbid condition that not only restricts erythropoiesis, but also disturbs the energy metabolism of the heart, kidneys or muscles [87]. The etiology of ID in chronic disorders (e.g., HF, chronic kidney disease or chronic obstructive pulmonary disease) is multifactorial, with potential contributing mechanisms including e.g., iron-poor diet, malabsorption, pro-inflammatory state, gastrointestinal bleeding (frequently subclinical) or interactions with certain medications (for example proton pump inhibitors) [81,88]. In patients with HF with reduced left ventricular ejection fraction, ID is highly prevalent and correlates with worse quality of life, lower exercise capacity and eventually increased long-term mortality [89,90,91,92]. Similar observations have been published for HF patients with preserved left ventricular ejection fraction. In such patients, ID is also common and relates to worse symptomatology, quality of life and exercise tolerance [93,94], but without unequivocal impact on long-term outcomes such as mortality or re-hospitalizations [93].

ID in patients with chronic disorders (such as HF) is diagnosed and replenished based on the assessment of iron parameters in peripheral blood only [95]. These easily accessible parameters such as serum ferritin, transferrin saturation index (TSAT) or soluble transferrin receptor (sTfR) indeed reflect to some extent iron status in peripheral tissues. For example, we have demonstrated that in patients with coronary artery disease (referred for surgical revascularization), elevated sTfR correlates with bone marrow iron depletion [96]. Analogously, in patients with HF the criterion of functional ID based on TSAT < 20% has been validated with ID confirmed in bone marrow iron staining [97]. It is also worth noting that in advanced HF myocardial and serum iron status are related to each other to some extent, e.g., circulating sTfR (indicator of iron demand in peripheral tissues) correlates with myocardial iron amount as well as with the myocardial expression of transferrin receptor, as assessed in explanted failing hearts [98]. This approach oriented at diagnosing and correcting the so-called “systemic ID”, as reflected by a few common iron parameters assessed in peripheral blood, is indeed clinically very practical, however, it does not provide any direct insight into what happens with the iron in particular organs on the cellular and sub-cellular level. Given that circulating indices of iron status predict clinical outcomes in chronic diseases, they have been targeted in clinical trials, for example in acute or chronic HF [14,99]. Nevertheless, more comprehensive understanding of iron homeostasis in particular organs in health and disease could translate into further benefits for patients. One may ask an intriguing question of whether there are patients with chronic disorders who would benefit from iron therapy through improving functioning of particular organs, in spite of indices of iron status in peripheral blood within the normal range.

Data on myocardial iron status in failing vs. non-failing human heart are limited. In advanced HF, myocardial iron content is reduced as it has been demonstrated in severely compromised hearts explanted before the transplantation procedure [15,17,59,98,100,101]. In one study it has been demonstrated that myocardial ID is common in advanced HF (explanted hearts) and is related to greater severity of left ventricular dysfunction and more symptomatic disease [15]. Importantly, predefined left ventricular myocardial ID correlated with decreased activity of CAC and respiratory chain activity as well as reduced anti-oxidant properties [15]. Once again, the above-cited study regarding direct tissue analysis of explanted failing hearts [59] should also be recalled, where it was shown that although myocardial ID was not associated with the activity of respiratory chain enzymes, it correlated with a decrease in important CAC enzymes activity (aconitase and citrate synthase) as well as with reduced expression of protective antioxidant enzymes. Diminished myocardial iron is also associated with greater remodeling—in one study regarding patients with non-ischemic cardiomyopathy hearts with low iron content were characterized by greater neurohormonal activation and myocardial remodeling, however, amount of cardiac iron was once again not related to levels of circulating iron biomarkers in these subjects [102].

There is evidence from experimental animals and cell cultures that the state of ID has detrimental impact on the performance of both the whole heart and isolated cardiomyocytes in vitro [103,104,105,106]. Experimentally, we can develop ID by introducing the specific low-iron diet for an animal or through chelating iron in the culture (environmental iron depletion), or alternatively we can “generate” ID within the whole organism or in selected organs/cells using precise knockouts of genes involved in iron metabolism (transgenic ID). It needs to be emphasized that in experimental animals the induction of a severe ID frequently leads to the development of anemia, and therefore the overlapping of effects of ID and decreased haemoglobin needs to be acknowledged [103,107]. Importantly, in a few studies iron was first limited and then supplemented, which confirms the causal relationship between ID and detected pathologies that were due successfully reversed [103].

**Figure 2:**
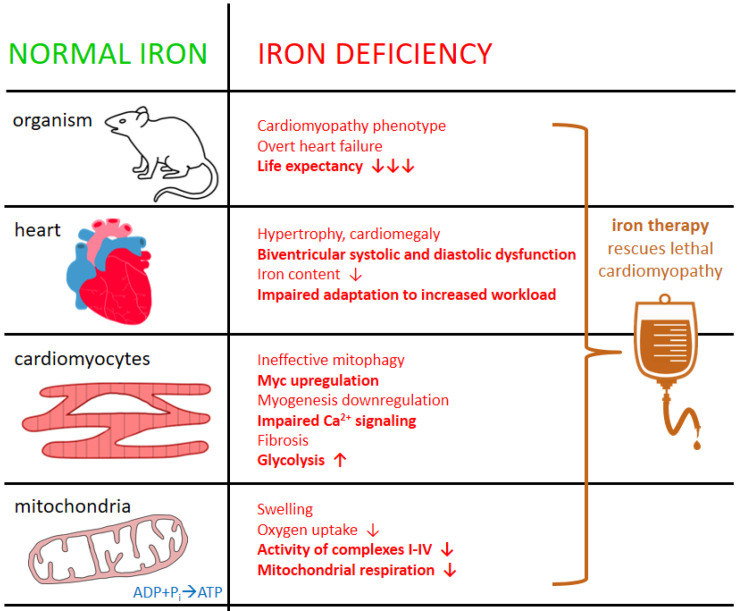
Detrimental effects of iron deficiency on the heart at various levels of tissue organization. For details and references—see Section 4.2.

Multifaceted abnormalities within animal hearts in the course of inducible ID are visible at all levels of tissue organization, from the entire organ, through isolated cardiomyocytes, ending with sophisticated mitochondrial pathobiology (Figure 2). Not surprisingly, rodents with ID have lower iron content in their hearts as compared with control animals [108]. As far as a few decades ago it has also been demonstrated that rats persistently fed with an iron-deficient diet presented with cardiac hypertrophy and myocardial mitochondria had decreased cytochrome content and activity with reduced overall oxygen uptake [109]. Indeed, dietary ID affects morphology of the heart (resulting in dilatation and hypertrophy, systolic compromise) and on the tissue level it enhances apoptosis [12,13,107,108,110,111]. Regarding cardiac hypertrophy as an initial morphological adaptation to mechanical overload of both the heart and cardiovascular system as a whole (finally leading to progressive heart failure), the differences between volume and pressure overload (responsible for concentric and eccentric hypertrophy, respectively) should be highlighted here [112,113]. Rats with anemia due to ID present with left ventricular dilatation and therefore as a model of HF development they represent eccentric rather than concentric hypertrophy [112]. The question of whether the broadly understood pathophysiology of ID (systemic and in particular body compartments/organs) in the course of HF caused predominantly by pressure overload (with initial adaptive concentric hypertrophy) is different compared to volume-overload HF, requires further experimental studies.

ID also influences isolated cardiomyocytes and their mitochondria, the latter both morphologically (altered structure) and functionally (reduced oxygen uptake, decreased activity of respiratory chain complexes) [12,104,105,106,109]. ID is also related to myocardial calcium signaling—one of emerging pathways attributed to impaired contractility of human failing hearts [107,114]. In one murine model animals with dietary IDA developed remarkable biventricular contractile dysfunction, which was accompanied by decreased cytoplasmatic Ca^2+^ transient amplitude in isolated cardiomyocytes [107]. Importantly, i.v. iron therapy with FCM reversed these abnormalities [107]. Another study also showed beneficial effects of i.v. iron on abnormal myocardial functioning due to ID—in rats made iron-deficient to induce adverse myocardial remodeling (dilated cardiomyopathy phenotype, fibrosis), intravenous iron sucrose reversed initial maladaptations along with the decrease in oxidative stress and pro-inflammatory and remodeling biomarkers within cardiac muscle [13]. With regard to isolated cardiomyocytes experimentally made iron-deficient, it has been demonstrated in one study that they present with impaired contractility along with decreased mitochondrial respiration, and these abnormalities were also reversed by adding iron back to the culture [115]. We have demonstrated that both during or without hypoxia, ID impairs the viability of rat cardiomyocytes [104,105]. Additionally, in human cardiomyocytes subjected to mechanical work decreased iron intensifies glycolysis and increases lactate production but mitochondrial aerobic metabolism is reduced [106].

Knockout murine models of ID restricted to cardiac muscle provide additional evidence on the critical role of adequate availability of iron for effective mechanical and energetic performance of cardiomyocytes [100,116]. With regard to methodology, myocardium-targeted deprivation of iron in transgenic animals is achieved either by directly affecting transferrin receptor or indirectly through modulating regulatory cascades (IRP). The latter maintain sufficient intracellular iron for synthesis processes and metabolic needs [100]. For example, in one study the authors blocked selectively myocardial iron uptake by inactivating transferrin receptor in cardiomyocytes [116]. Consequently, experimental animals did not increase their cardiac iron throughout an early postnatal period (due to constantly deranged iron uptake from circulation) and shortly demonstrated the entire spectrum of severe morphological and functional abnormalities in their hearts, including cardiomegaly, hypertrophy, impaired contractile function, enlarged mitochondria, ineffective mitophagy, and eventually the decreased activity of electron transport chain complexes [116]. Finally, messenger ribonucleic acid signatures within the hearts of transgenic mice demonstrated the upregulation of Myc (gene playing key role in cell lifecycle and survival), glycolytic and hypoxia-inducible factor genes, and the downregulation of genes involved in myogenesis and peroxisome proliferator-activated receptor, peroxisome proliferator-activated receptor gamma coactivator 1-alpha and insulin signaling [116]. Importantly, an aggressive iron replenishment in severely cardiac iron-deficient animals inhibited to some extent the development of aforementioned lethal cardiomyopathy [116]. In another study of Haddad et al. [100] cardiomyocyte-specific deletion of IRP in mice resulted in the decrease in cardiomyocyte iron content, impaired cardiac adaptation to acutely or chronically increased workload (during dobutamine test or in the model of progressive ischemic HF, respectively), and completely ineffective increase in mitochondrial respiration during exercise. These maladaptations were later reversed by FCM in knockout but not control animals [100]. A key body iron regulator hepcidin (mainly synthesized in the liver; it controls iron homeostasis in a negative feedback mechanism through inhibiting ferroportin) is also expressed in cardiac muscle, where its gene (hepcidin antimicrobial peptide gene—HAMP) can also be selectively deleted [117]. Namely, in the mouse model of cardiomyocyte-specific deletion of HAMP, the animals developed lethal cardiomyopathy with remarkable mitochondrial dysfunction and metabolic impairment [117]. Importantly, genetic modifications did not affect circulating iron biomarkers and cardiomyopathy due to cardiac ID was prevented with intravenous iron therapy analogously to previous experiments [117]. A similar phenotype of HF was observed in the second arm of the experiment, where cardiac-specific knock-in of hepcidin-resistant ferroportin was introduced demonstrating a unique model of uncontrolled iron release from cardiomyocytes without an autocrine control with cardiac hepcidin [117]. Nevertheless, a few studies have also demonstrated detrimental effects of iron overload on mitochondrial functioning with the subsequent development of cardiac compromise, e.g., mice with inducible mitochondrial iron overload (ATP binding cassette transporter B8 knockout) develop cardiac systolic and diastolic dysfunction, along with the increased oxidative stress and structural abnormalities within mitochondria [118]. Indisputably, precise control mechanisms of iron homeostasis in the heart protect against both harmful deficiency and excess of this micronutrient.

## 5. Conclusions

Impaired myocardial energetics constitutes an important pathophysiological feature of HF at molecular level and is considered to promote clinical progression of the disease, not infrequently in spite of administered evidence-based cardioprotective treatments [2,4,5]. Iron plays a number of important biochemical roles in the cell, and bidirectional abnormalities regarding iron homeostasis (excess vs. deficiency) negatively affect cell biology and viability [62,63,65]. It has been demonstrated in a few well-designed translational studies that ID adversely affects the functioning of the heart at different levels of tissue organization (including leading to its overt failure), and iron supplementation reverses at least some of these maladaptations [103]. There is limited evidence that in the course of its advanced failure (before transplantation) the human heart is also often deficient in this micronutrient, which is to some extent related to unfavorable remodeling, energetics and patient clinical status [15,17,59,98,100,101]. There are published a few clinical trials demonstrating that the correction of systemic ID (diagnosed with biomarkers from blood sample) in HF results in the improvement of quality of life, symptoms and exercise capacity, and these effects go beyond simply improving red cell indices [81,119]. Moreover, clinical benefits in terms of reducing HF hospitalizations (and therefore affecting the natural history of the disease) have been demonstrated in iron-deficient patients stabilized after an episode of acute HF who were treated pre-discharge with FCM (intravenous iron) [14]. It needs to be acknowledged, however, that we have no data for if and how intravenous iron supplementation in patients with HF and systemic ID (assessed in peripheral blood) affects iron levels in the heart. Interestingly, there are scarce data suggesting that intravenous iron load can affect skeletal muscle energetics in iron-deficient subjects with HF [11]. It is reasonable to further study systemic effects of (intravenous) iron therapy on individual iron compartments in the body, including myocardial iron levels, especially in the context of possible metabolic improvement potentially translating into reduced morbidity.

## Data Availability

Not applicable.

## Abbreviations

AMPK	adenosine monophosphate-activated protein kinase
ATP	adenosine triphosphate
CAC	citric acid cycle
CK	creatine kinase
FAs	fatty acids
FCM	ferric carboxymaltose
HAMP gene	hepcidin antimicrobial peptide gene (encoding hepcidin)
HF	heart failure
ID	iron deficiency
IRP	iron regulatory proteins
LIP	labile iron pool
PCr	phosphocreatine
ROS	reactive oxygen species
sTfR	soluble transferrin receptor
TSAT	transferrin saturation (index)
